# Development and Implementation of PHC‐CST: A Cognitive Screening Tool for Early Detection of Dementia in Primary Health Care Settings in India

**DOI:** 10.1002/alz70860_096673

**Published:** 2025-12-23

**Authors:** Jeevitha G R

**Affiliations:** ^1^ M S Ramaiah University of Applied Sciences, Bangalore, Karnataka, India

## Abstract

**Background:**

The increasing prevalence of dementia in India highlights the need for effective and accessible diagnostic tools in primary health care. Existing cognitive screening tools face cultural and logistical challenges, reducing their applicability in diverse settings. The objective of the study was to develop, validate, and evaluate the Primary Health Care Cognitive Screening Tool (PHC‐CST) for the early detection of dementia, tailored specifically for use in resource‐limited settings in India.

**Methods:**

A single‐stage study involving 97 participants aged 50 years and older was conducted at the Kaiwara Primary Health Care Centre in Karnataka, India. The PHC‐CST was designed through a multi‐phase process, involving extensive input from stakeholders, including nurses, doctors, and patients, to ensure cultural and contextual relevance. Validation measures included sensitivity, specificity, and inter‐rater reliability, benchmarked against the Montreal Cognitive Assessment (MoCA). The tool features simplified language, contextually relevant tasks, and a scoring system adapted to local demographics.

**Results:**

The PHC‐CST demonstrated strong validity, with a sensitivity of 89% and specificity of 85% for detecting early dementia. Stakeholders reported high ease of use, minimal training requirements, and seamless integration into existing workflows. Compared to MoCA, PHC‐CST improved diagnostic accuracy and reduced administration time. Qualitative feedback highlighted its cultural relevance and scalability in similar settings.

**Conclusion:**

The PHC‐CST addresses critical gaps in dementia diagnosis within primary health care settings in India. Its culturally tailored design, ease of use, and robust diagnostic performance position it as a promising tool for early dementia detection, with the potential for broader application in low‐resource environments.

